# Hypervolemia increases release of atrial natriuretic peptide and shedding of the endothelial glycocalyx

**DOI:** 10.1186/s13054-014-0538-5

**Published:** 2014-10-13

**Authors:** Daniel Chappell, Dirk Bruegger, Julia Potzel, Matthias Jacob, Florian Brettner, Michael Vogeser, Peter Conzen, Bernhard F Becker, Markus Rehm

**Affiliations:** Department of Anesthesiology, University Hospital of Munich, Marchioninistrasse 15, 81377 Munich, Germany; Department of Clinical Chemistry, University Hospital of Munich, Marchioninistrasse 15, 81377 Munich, Germany; Walter-Brendel Centre of Experimental Medicine, Ludwig-Maximilians-University Munich, Schillerstrasse 44, 80336 Munich, Germany

## Abstract

**Introduction:**

Acute normovolemic hemodilution (ANH) and volume loading (VL) are standard blood-sparing procedures. However, VL is associated with hypervolemia, which may cause tissue edema, cardiopulmonary complications and a prolonged hospital stay. The body reacts to hypervolemia with release of atrial natriuretic peptide (ANP) from the heart. ANP has been shown to deteriorate the endothelial glycocalyx, a vital part of the vascular permeability barrier. The aim of the present study was to evaluate and compare ANP release and damage to the glycocalyx during ANH and VL.

**Methods:**

ANH or VL with 6% hydroxyethyl starch 130/0.4 was administered prior to elective surgery in patients of good cardiopulmonary health (*n* =9 in each group). We measured concentrations of ANP in plasma and of three main constituent parts of the glycocalyx (hyaluronan, heparan sulfate and syndecan 1) in serum before and after ANH or VL. Heparan sulfate and syndecan 1 levels in urine were also determined.

**Results:**

In contrast to ANH, VL (20 ml/kg) induced a significant release of ANP (approximately +100%, *P* <0.05) and increased the serum concentration of two glycocalyx constituents, hyaluronan and syndecan 1 (both by about 80%, *P* <0.05). Elevation of syndecan 1 was also detected in the urine of patients undergoing VL, but no increase was found in patients undergoing ANH. Heparan sulfate levels were not influenced by either procedure.

**Conclusion:**

These data suggest that hypervolemia increases the release of ANP and causes enhanced shedding of the endothelial glycocalyx. This perturbation must be expected to impair the vascular barrier, implying that VL may not be as safe as generally assumed and that it should be critically evaluated.

## Introduction

On the surface of healthy vascular endothelium resides a structure, the endothelial glycocalyx, that consists of extracellular domains of receptor, adhesion and channel proteins and, foremost, of molecules such as transmembrane syndecan 1 (bearing covalently bound, highly negatively charged glycosaminoglycans, such as heparan and chondroitin sulfates) and the receptor-bound, long-chained hyaluronic acid molecule [1,2]. Together with bound plasma proteins and glycosaminoglycans, the glycocalyx forms the endothelial surface layer with a functional thickness of more than 1 μm [3,4]. Disruption of the glycocalyx has been shown to increase capillary permeability and firm attachment of leukocytes and blood platelets, leading to tissue edema, suggesting that the glycocalyx acts as a competent permeability barrier and antiadhesive interface with blood [5]. In patients in septic shock, glycocalyx shedding has been found to be associated with increased mortality and to be an independent predictor of mortality in trauma patients [6–8].

Traditional perioperative fluid therapy is often performed in a liberal manner, leading to fluid overload and tissue edema. In clinical studies, hypervolemia has been shown to have detrimental influences on several aspects of patient outcome, including cardiopulmonary complications, anastomotic insufficiency, length of hospital stay, duration of mechanical ventilation and mortality [9–11]. One possible underlying trigger may be the release of atrial natriuretic peptide (ANP) from the cardiac atria invoked by mechanical wall stress [12]. ANP is known to induce rapid shifts of intravascular fluid into the interstitial space and has been shown to cause deterioration of the endothelial glycocalyx. In an isolated heart model, artificially infused ANP induced an increase in vascular permeability, a histologically detectable degradation of the glycocalyx and significant tissue edema [13]. Furthermore, an increase in plasma ANP has been found to precede shedding of the glycocalyx in patients undergoing coronary bypass surgery [14].

ANP is released during hypervolemia, which could explain the context sensitivity of volume effects of iso-oncotic colloidal infusions (for example, 6% hydroxyethyl starch or 5% human albumin) [15,16]. The “volume effect” is that part of an infused fluid bolus that remains within the vasculature for a longer period. Direct blood volume measurements have revealed that the presumed volume effect of about 100% for such colloids is realized only in normovolemic patients—that is, during acute normovolemic hemodilution (ANH), a procedure in which blood is withdrawn and simultaneously replaced by equal amounts of colloidal fluid [16]. During volume loading (VL) (that is, the hypervolemic infusion of colloids into a primarily normovolemic circulation without simultaneous blood withdrawal), about 60% of the infused amount was observed to directly load the interstitial space within 30 minutes, leaving a volume effect of merely 40% [15]. Also, 60% of the infused fluid was found to leave the intravascular space, and 60% of the administered macromolecules (hydroxyethyl starch (HES) and albumin) were extravasated.

The aim of the present study was to investigate comparatively whether the established blood-sparing procedures VL (hypervolemia) and ANH (normovolemia) are associated with increased ANP levels and shedding of the endothelial glycocalyx.

## Material and methods

The study was approved by the independent ethics committee of our institution (Medical Faculty of the Ludwig-Maximilians-University Munich, Germany; trial registration 128-04), and all patients included gave us their written informed consent to participate. Inclusion criteria were patient consent, good cardiopulmonary health, expected blood loss of more than 500 ml, and preoperative indication of the necessity to insert arterial and central venous catheters. The study was performed before the results of the 6S and CHEST trials were published (Scandinavian Starch for Severe Sepsis/Septic Shock trial and Crystalloid versus Hydroxyethyl Starch trial, respectively) [17,18].

### Anesthesiological procedure

The mandatory fasting period after consumption of solid food, milk and milk-containing fluids was 6 hours, and clear fluids were allowed up to 2 hours before induction of anesthesia. After each patient arrived in the operating theatre, monitoring with electrocardiography and pulse oximetry was implemented. General anesthesia was induced with 0.5 μg/kg sufentanil, 2 mg/kg propofol and 0.1 mg/kg cisatracurium, and, after tracheal intubation, anesthesia was maintained with 1.6% to 2.0% sevoflurane. Radial artery and central venous catheters were inserted. Mechanical ventilation was performed to maintain partial pressure of arterial oxygen at 100 to 150 mmHg and partial pressure of carbon dioxide at 40 ± 5 mmHg. No intravenous infusions were applied, except for negligible amounts required to inject the intravenous drugs. After a steady state was achieved, the patients were assigned to receive either VL or ANH.

### Acute normovolemic hemodilution

Blood was removed at a rate of about 60 ml/min via the arterial line and simultaneously replaced with iso-oncotic HES colloid solution (6% HES 130/0.4 (Volulyte); Fresenius Kabi, Bad Homburg, Germany) at almost the same rate through the central venous line. At first, a quantity of approximately 500 ml/m^2^ of blood was removed. The hemodilution bags were weighed on a precision scale so that the volume of withdrawn blood could be evaluated immediately. For fine-tuning, frequent determinations of hematocrit were carried out to reach a target hematocrit level of 24 ± 2%. The hemodilution procedure took about 30 minutes.

### Volume loading

VL was initiated by infusing 20 ml/kg of an iso-oncotic HES colloid solution (6% HES 130/0.4, Volulyte) within 15 minutes at a rate of approximately 90 ml/min.

### Blood sampling

In all patients, blood and urine samples were taken directly after induction of anesthesia (pre) to determine basal values. Both VL and ANH procedures were initiated immediately thereafter and lasted for 30 minutes, followed by the second blood and urine sampling (post). The time window for the present study was chosen because we knew from previous direct double-tracer blood volume measurements that, after VL with 5% human albumin or 6% HES administration, the total volume of the endothelial surface layer (glycocalyx and bound plasma proteins) would be significantly reduced 30 minutes after the procedure [15]. The patients were kept strictly in supine position during the whole investigation period. All measurements were performed before surgical incisions were made.

### Determination of glycocalyx constituents

Immediately after withdrawal into serum vials, blood samples were centrifuged at 2,000 *g* for 10 minutes. The serum fraction was frozen and stored at −80°C until assayed. Urine samples were also frozen at −80°C until assayed.

### Syndecan 1 concentration

Syndecan 1 concentrations in serum and urine were determined directly as previously reported by using an enzyme-linked immunosorbent assay kit (Diaclone Research, Besancon, France) [14,19,20]. In this kit, a solid-phase monoclonal B-B4 antibody against an extracellular domain of syndecan 1 is employed.

### Heparan sulfate concentration

Serum and urinary heparan sulfate concentrations were quantified using a special enzyme-linked immunosorbent assay kit (Seikagaku, Tokyo, Japan) as previously reported [14,19,21–23]. Serum was pretreated with protease from *Streptomyces griseus* (Actinase E; Sigma-Aldrich, St Louis, MO, USA). This kit employs two monoclonal antibodies specific for heparan sulfate–related epitopes.

### Hyaluronan concentration

Hyaluronan concentrations were measured in serum using an enzyme-linked immunosorbent assay kit (Echelon Biosciences, Salt Lake City, UT, USA) designed to detect human hyaluronic acid, as previously described [14,23]. Hyaluronan concentrations were not measured in urine, because renal excretion plays a negligible part in clearance. The major part of the elimination of hyaluronan (about 90%) from the systemic circulation occurs in the liver via receptor-mediated endocytosis in the sinusoidal liver endothelial cells [24,25].

### Atrial natriuretic peptide concentration

Blood was sampled into ethylenediaminetetraacetic acid in vials and centrifuged immediately (2,000 *g*, 10 minutes). The plasma was directly frozen and stored at −80°C until assayed. Plasma levels of ANP were measured with a sandwich enzyme-linked immunosorbent assay kit (Uscn Life Science, Wuhan, China) with an antibody specific to human ANP as performed previously [14]. The kit did not require a preliminary step for extraction or purification of plasma samples.

### Statistical analysis

A preliminary sample size calculation was based on a previous study in which researchers investigated ANP concentrations after VL with 500 ml of 6% HES and 1,000 ml of Ringer’s lactate solution in patients before caesarean section [26]. The difference between mean ANP before and after VL was 17.9 pg/ml. Using this difference and the corresponding standard deviations, we calculated a sample size with a two-sided confidence interval of 0.95 and a desired power of 0.80, which yielded a result that we needed nine patients in each group. Data are given as mean ± SEM. All data were compared by performing Student’s *t*-test, with *P* <0.05 considered statistically significant (SigmaStat; Systat Software, Richmond, VA, USA).

## Results

### Demographic data and procedural characteristics

All patients were attested to be in good cardiopulmonary health and were scheduled for elective general surgery with an expected blood loss greater than 500 ml. As the measuring time points were all before skin incision, the individual diagnosis and operation had no influence on the study. The pertinent patient characteristics and initial albumin concentrations are listed in Table 1. Patients in the VL group received 1,326 ± 50 ml of 6% HES solution after induction of anesthesia. In the ANH group, 1,267 ± 62 ml of blood were drawn and simultaneously replaced with similar amounts of 6% HES 130/0.4. Notably, urine production was not increased in the VL group compared to the ANH group during the first 30-minute period of the intervention.

**Table 1:** **Patient characteristics** ^**a**^

	**VL**	**ANH**
Age (yr)	57 ± 4	55 ± 2
Height (cm)	170 ± 2	166 ± 2
Weight (kg)	67 ± 3	64 ± 4
BSA (m^2^)	1.8 ± 0.1	1.7 ± 0.1
Blood removed (ml)		1,267 ± 62
Colloid infused (ml)	1,326 ± 50	1,298 ± 71
Urine production (ml)	213 ± 66	228 ± 29
Baseline serum albumin (g/dl)	4.1 ± 0.1	4.1 ± 0.1

^a^Values are given as mean ± SEM. There were no significant differences between the VL and ANH groups with respect to applicable parameters. Urine production was determined 30 minutes after fluid infusion (VL) or 30 minutes after blood draw (ANH), respectively. ANH, Acute normovolemic hemodilution; BSA, Body surface area; VL, Volume loading.

### Atrial natriuretic peptide

**Figure 1:**
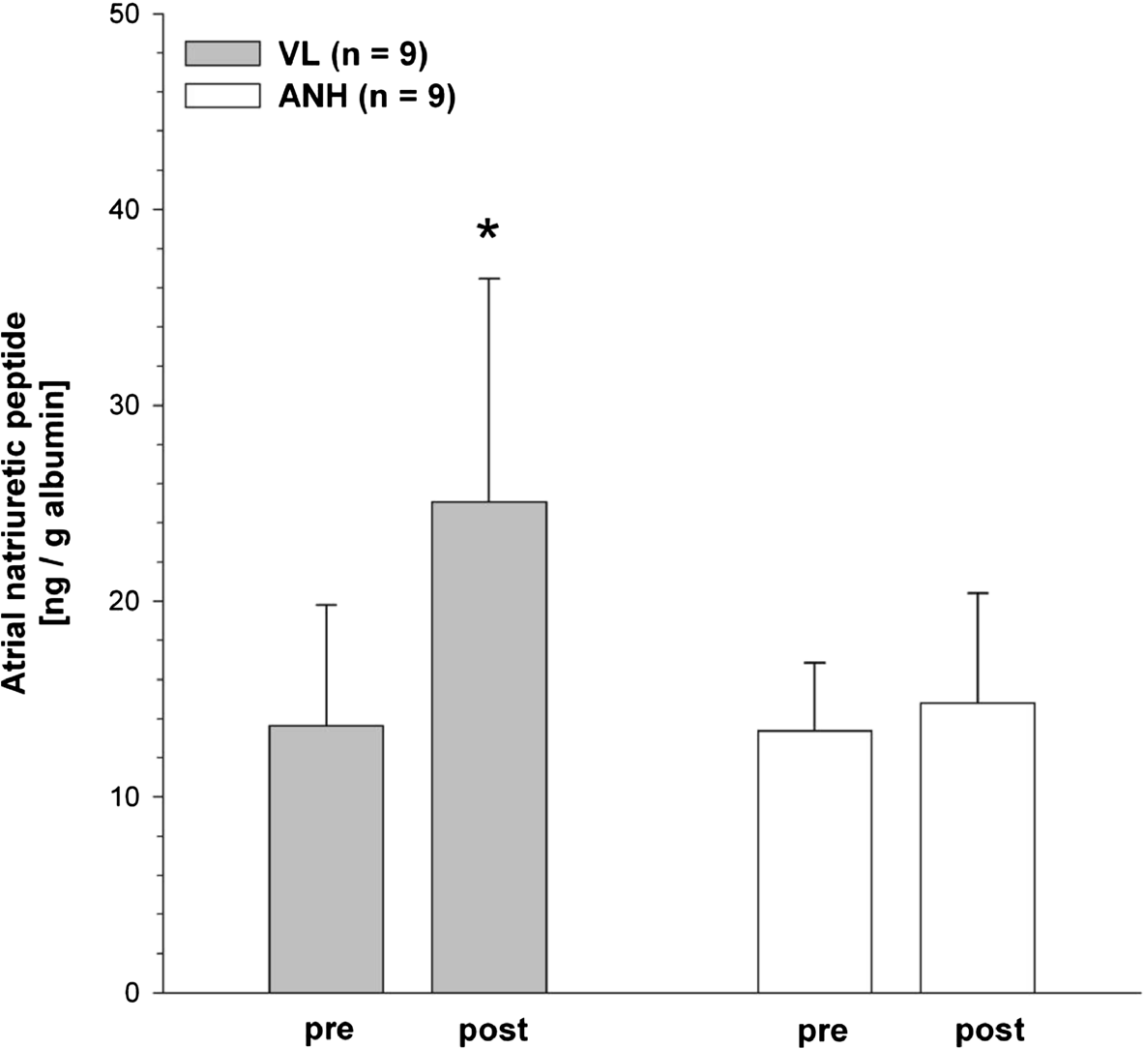
**Atrial natriuretic peptide concentrations.** Atrial natriuretic peptide concentrations before (pre) and after (post) volume loading (VL, *n* =9) or acute normovolemic hemodilution (ANH, *n* =9) with 6% hydroxyethyl starch 130/0.4. To account for hemodilution, individual atrial natriuretic peptide concentrations were normalized to the individual albumin concentrations. Data are given as mean ± SEM. *P <0.05 (significantly different from pre).

All data are normalized to grams per deciliter of plasma albumin to account for varying degrees of hemodilution in individual patients. Basal ANP concentrations in plasma showed no difference between the VL and ANH groups (13.6 ± 6.2 ng/g vs. 13.4 ± 3.5 ng/g albumin). Thirty minutes after the procedures, ANP concentrations had increased in the VL group to 25.1 ± 11.4 ng/g albumin, whereas the ANP concentration in the ANH group remained almost unchanged at 14.8 ± 5.6 ng/g albumin (*P* <0.05) (Figure 1).

### Shedding of components of the endothelial glycocalyx

**Figure 2:**
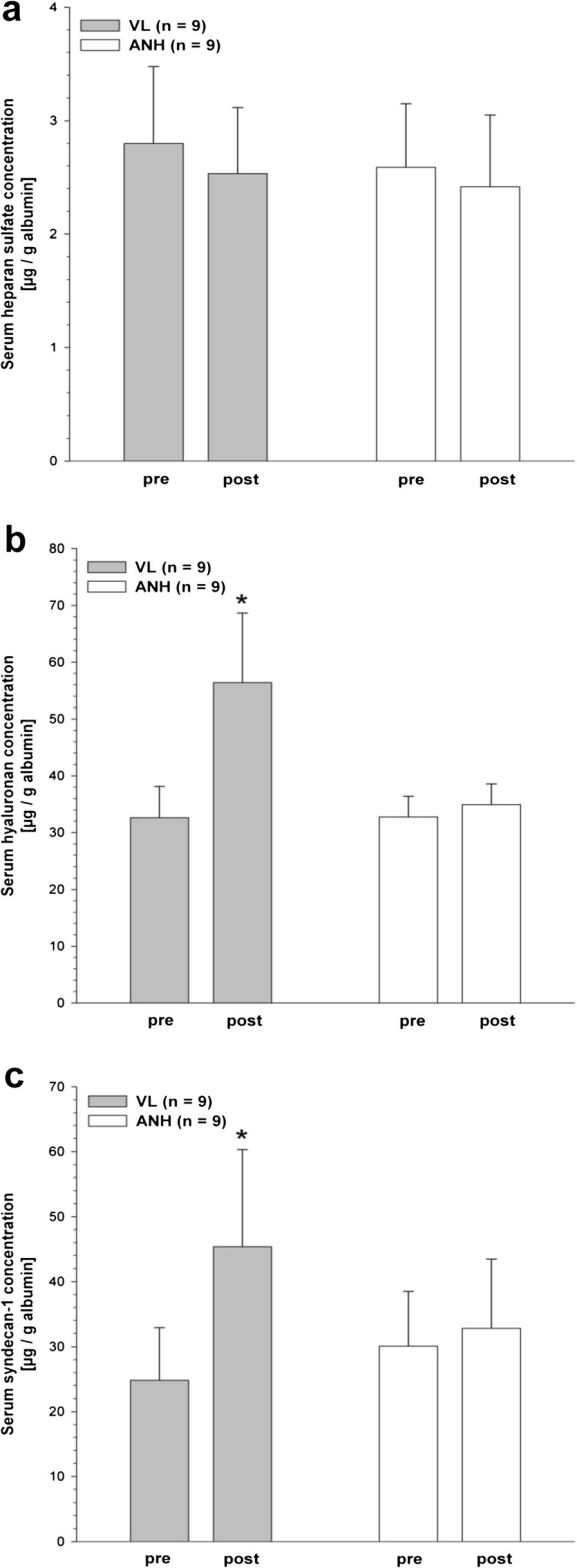
**Serum concentrations of glycocalyx components.** Serum concentrations of heparan sulfate **(a)**, hyaluronan **(b)** and syndecan 1 **(c)** before (pre) and after (post) volume loading (VL, *n* = 9) or acute normovolemic hemodilution (ANH, *n* = 9) with 6% hydroxyethyl starch 130/0.4. To account for hemodilution, individual heparan sulfate, hyaluronan and syndecan 1 concentrations were normalized to the individual albumin concentrations. Data are given as mean ± SEM. *P <0.05 (significantly different from pre).

To account for hemodilution, individual heparan sulfate, hyaluronan and syndecan 1 concentrations were normalized to the individual albumin concentrations. Respective baseline values of heparan sulfate, hyaluronan and syndecan 1 concentrations did not differ between the groups (heparan sulfate: 2.8 ± 0.7 μg/g albumin vs. 2.6 ± 0.6 μg/g albumin; hyaluronan: 32.6 ± 5.5 μg/g albumin vs. 32.7 ± 3.7 μg/g albumin; syndecan 1: 24.8 ± 8.1 vs. 30.1 ± 8.4 μg/g albumin) (Figure 2). Figure 2 illustrates the changes in the concentrations of the three glycocalyx constituents in both groups. Serum heparan sulfate concentrations remained almost unchanged after both VL and ANH (Figure 2a). Interestingly, the serum hyaluronan concentration increased significantly to 56.4 ± 12.2 μg/g albumin in the VL group 30 minutes after the procedure, whereas the level remained unchanged in the ANH group (Figure 2b). Also, a significant increase in serum syndecan 1 concentration to 45.4 ± 14.9 μg/g albumin was found in the VL group 30 minutes after VL. Again, no change in serum syndecan 1 was seen 30 minutes after ANH (Figure 2c).

### Renal excretion of components of the endothelial glycocalyx

**Figure 3:**
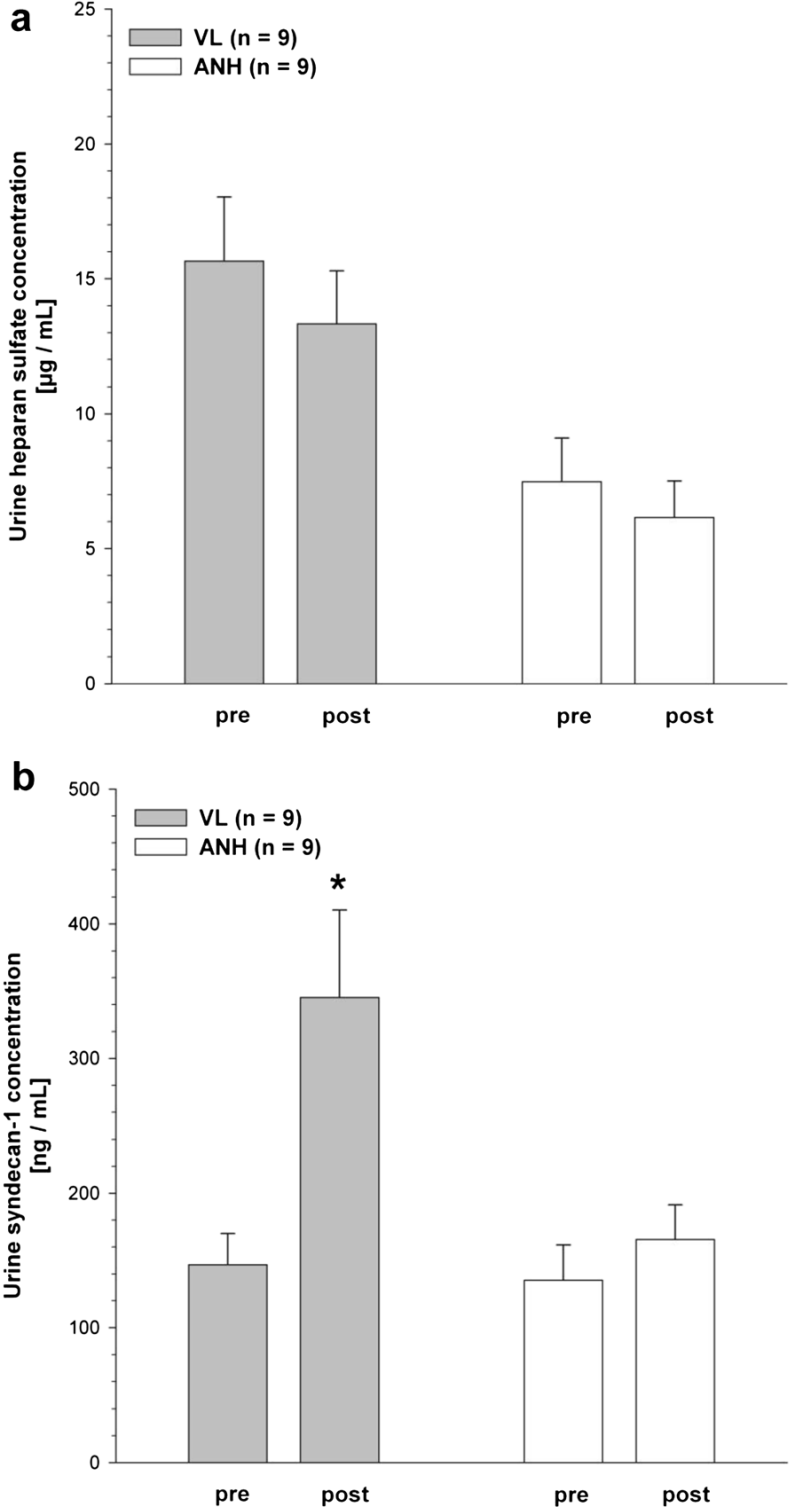
**Urinary concentrations of glycocalyx components.** Urinary concentrations of heparan sulfate **(a)** and syndecan 1 **(b)** before (pre) and after (post) volume loading (VL, *n* = 9) or acute normovolemic hemodilution (ANH, *n* = 9) with 6% hydroxyethyl starch 130/0.4. Data are given as mean ± SEM. *P <0.05 (significantly different from pre).

Figure 3 shows the changes in urinary concentrations of two glycocalyx components known to pass through the glomerular barrier. Urinary heparan sulfate concentrations remained unchanged both after VL and after ANH (Figure 3a). In contrast, urinary syndecan 1 concentrations increased significantly in the VL group 30 minutes after VL (*P* <0.05). No such increase was found in the ANH group (Figure 3b).

## Discussion

The results of the present study reveal that hypervolemia induced by VL is associated with increased plasma concentrations of ANP and raised serum levels of two main constituents of the endothelial glycocalyx. ANH had no comparable effects.

ANH and VL are well-established, frequently used blood-sparing procedures. The main goal of ANH is to withdraw whole blood, which is stored outside the body, and replace this volume with iso-oncotic colloids, thereby diluting the remaining blood whilst retaining a constant normovolemic blood volume [27]. In this situation, iso-oncotic colloids have a volume effect of nearly 100%, meaning that the entire infused volume remains in the circulation [16]. In the event of subsequent bleeding, there is a loss of diluted, hemoglobin-reduced blood, which can be replaced with the previously withdrawn hemoglobin-rich blood when the individual transfusion cutoff value is reached. VL, in contrast, dilutes the blood by expanding the blood volume [27]. In previous studies, direct blood volume measurements have revealed that, during the VL procedure, only 40% of the infused colloids remain within the vasculature [15]. The other 60% are shifted to the interstitial space, causing tissue edema. The mechanism behind this colloid shifting seems to be an impaired glycocalyx structure, as evidenced by a considerable decrease in the total volume of the endothelial surface layer [15]. The results of the present study offer an explanation for this phenomenon: ANP is released from human atria during iatrogenic hypervolemia, inducing shedding of constituent parts of the endothelial glycocalyx.

ANP has well-known diuretic, natriuretic and vasodilating effects. Furthermore, this polypeptide hormone also induces rapid shifts of intravascular fluid into the interstitial space [12,28]. Experimental and clinical studies have revealed that elevation of ANP is associated with increased shedding of the endothelial glycocalyx [13,14].

A possible connection between the ability of ANP to acutely shift volume from the intra- to extravascular spaces and its influence on the integrity of the endothelial glycocalyx has been investigated in an experimental setting by our group [13]. Infusion of ANP in an isolated heart model caused an increase in fluid leak and an accelerated extravasation of colloid. Furthermore, ANP resulted in rapid shedding of the endothelial glycocalyx and histologically detectable destruction of the coronary vascular glycocalyx [13]. Accordingly, the ANP-induced increase in vascular permeability described *in vivo* might be related to changes in the integrity of the glycocalyx. This fragile structure is a crucial part of the vascular barrier, and its deterioration has been shown to cause shifting of fluids and colloids into the interstitial space with concomitant tissue edema.

Indirect evidence for such an effect comes from a recent study of patients undergoing coronary artery bypass surgery [14]. Three major components of the endothelial glycocalyx were shed from the endothelium and detected in the circulating blood of patients during both on- and off-pump coronary artery bypass surgeries. Comparison of the respective time courses of glycocalyx shedding with release of ANP and various cytokines designated ANP as the mediator that most likely initiated shedding in both surgical procedures [14].

Perioperative fluid therapy is currently being discussed intensively concerning not only the kind of fluid but also the right amount. A further aspect of debate is when to infuse the fluid. The danger of hypovolemia’s causing organ hypoperfusion and increasing the risk of systemic inflammation has been known for decades. However, awareness that hypervolemia can have similar detrimental effects on patient outcomes is relatively new. Fluid overload has been shown to abet anastomotic leakage, pulmonary edema, pneumonia and wound infection, as well as postoperative ileus [10,11,29]. Individually, both the surgical impact (inflammation, trauma) and the traditional liberal fluid strategy have the potential to impair the competence of the vascular barrier. The combination of both confounders increases this effect significantly. A breakdown of vascular barrier competence results in capillary leakage and protein-rich interstitial edema, which lead to a significant perioperative increase in body weight. In clinical trials, this has been shown to be associated with increased morbidity and mortality [9–11]. The data derived from the present study support the need for a rational fluid management strategy to maintain normovolemia by replacing actual losses in an adequate manner and avoid unnecessary fluid boluses.

HES preparations are frequently used in clinical practice because their intravascular volume effect is high and their deposition in tissue is low. Under pathological conditions, however, this might not inevitably be the case. Patients with severe sepsis and in septic shock, for example, need very large amounts of fluid to stabilize cardiac preload. Trials have revealed a significantly decreased volume effect of colloids in this situation, despite remaining superior to crystalloids [30–33]. This effect not only is associated with severe tissue edema but also can cause serious side effects that have a negative impact on patient outcome. Experimental data support the observation that colloids may be retained insufficiently at the vascular barrier during capillary leakage [34]. Our experimental findings are in agreement with these clinical observations linking degradation of the glycocalyx to colloid and fluid extravasation.

One key question is whether the renal or the vascular effects of ANP stand in the foreground. In our study, we measured no difference in urinary output between these groups, although patients in the VL group were subjected to hypervolemia. However, our urine-sampling period terminated directly after fluid infusion and thus was probably too short to detect changes in urinary output. Indeed, Drummer *et al*. demonstrated that an intravenous volume bolus of 2 L in healthy volunteers required more than 48 hours to be completely excreted [35]. Nevertheless, the unchanged urine production in our study allowed us to analyze clearly the results obtained for urinary elimination of heparan sulfate and syndecan 1. The urinary concentration of the former did not differ between pre- and postintervention for either the VL or ANH patients, whereas the syndecan 1 concentration increased in the VL group. Thus, urinary elimination directly reflects the alteration in blood level determined for these two glycocalyx constituents, with no masking or simulation of shedding on the basis of renal clearance.

One limitation of this trial is that biomarkers of glycocalyx shedding are only an indirect method of measuring glycocalyx thickness and integrity and do not provide insight into the thickness of the remaining glycocalyx structure. Furthermore, we used a third-generation balanced 6% hydroxyethyl starch 130/0.4 solution, so, despite volume effects similar to those seen with other iso-oncotic colloids (for example, 5% human albumin), the results of this trial are not directly transferable to other colloids.

Surprisingly, we found only two markers of glycocalyx degradation to be elevated in the serum of the VL group: syndecan 1 and hyaluronan. It should be borne in mind that shedding of these two constituents requires the action of proteases, whereas the lyase heparanase is needed for shedding of heparan sulfates in humans. Accordingly, VL cannot lead to any marked activation or release of heparanase in humans. A major store of heparanase in humans is in the tissue mast cells [36]. Fittingly, there are no receptors for ANP on the human mast cell. Thus, VL-induced liberation of ANP is a realistic mechanism by which to explain our finding of dissociated markers of glycocalyx damage.

## Conclusion

The results of our trial show that the transfusion-sparing procedure of VL may not be as safe as believed. Hypervolemia has detrimental effects on vascular barrier competence and may impair patient outcome. We recommend abstaining from preoperative volume boluses and favoring ANH should hemodilution be warranted to reduce perioperative red cell transfusion. Future research in general is warranted in the area of fluid administration that takes the glycocalyx into consideration and includes deducing optimal volume amounts and rates of infusion that will maintain intravascular volume without causing disruption of the endothelial glycocalyx.

## Key messages

- ANP is released during preoperative VL.
- VL causes increased shedding of the endothelial glycocalyx.
- Glycocalyx components can be detected in both serum and urine 30 minutes after VL.
- ANH has much lower effects on both glycocalyx shedding and ANP release, respectively.

## Abbreviations

ANH: Acute normovolemic hemodilution
ANP: Atrial natriuretic peptide
BSA: Body surface area
HES: Hydroxyethyl starch
VL: Volume loading
